# Root canal retreatment of root canals filled with “Neo MTA 2” and epoxy resin, using continuous and asymmetric reciprocal rotation systems: *In vitro* study

**DOI:** 10.4317/jced.61266

**Published:** 2024-02-01

**Authors:** Alejandra Rodriguez-López, Keilin Moreno-Chavez, Elisabet Mingo-Zambrano, Maria Noguera-García, Maria-Lluïsa Ballester-Palacios, Esther Berástegui

**Affiliations:** 1 DDS. Master in Advanced and Experimental Endodontics. University of Barcelona, Spain; 2 DDS. Professor on Master in Advanced and Experimental Endodontics. University of Barcelona, Spain; 3 PhD, DMD, DDS. Professor on Master’s Advanced and Experimental Endodontics. University of Barcelona. Researcher of the Idibell Institute; 4 PhD, DMD, DDS. Director of Master’s Advanced and Experimental Endodontics. University of Barcelona. Researcher of the Idibell Institute

## Abstract

**Background:**

The study aimed to compare the amount of extrusion, the time taken to achieve apical patency and duration for removing filling material during the retreatment of teeth filled with NeoMTA 2® (Avalon Biomed, Houston, TX, USA), a calcium silicate hydraulic cement and AHPlus™ (Denstsply, Konstanz, Germany), a resin-based cement, using continuous rotation and asymmetric reciprocating rotation instruments.

**Material and Methods:**

60 monoradicular human teeth were selected and instrumented with Race #35.06 (FKG Dentaire, La Chaux-de-Fonds, Switzerland). The teeth were randomly assigned to two groups based on the cement used for filling (n = 30 each) AHPlus™ and NeoMTA 2®. Both groups were obturated with gutta-percha, using the lateral condensation technique. During non-surgical retreatments, each group was further divided into two subgroups: D-Race (FKG) and Reciproc (VDW, Munich, Germany) (n = 15 each). The total time to achieve apical patency and complete the retreatment were recorded. Extruded debris were collected and weighted. Student t-tests were employed for mean comparisons to assess significant differences between variables.

**Results:**

No statistically significant differences were found in terms of type of cement and apical extrusion. Regarding the rotation system, using the Reciproc system resulted in longer time to achieve patency and retreatment times compared to D-Race system, with statistically significant differences (*P*< 0.05).

**Conclusions:**

All systems used in retreatment led to apical extrusion. Apical patency was achieved in all cases.

** Key words:**D-Race, Reciproc, calcium silicate-based cement, root canal retreatment.

## Introduction

The objective of obturation in endodontics is to achieve the three-dimensional hermetic sealing of the root canal system, preventing the passage of microorganisms (1). Gutta-percha and sealer cements are commonly used for obturation. Recently, sealer cements based on calcium silicate have emerged, characterized by the formation of calcium hydroxide and the release of calcium ions. These cements offer dimensional stability, expansion during setting, excellent canal sealing and a pH greater than 12 (2). NeoMTA2® cement is composed of tricalcium silicate, dicalcium silicate, calcium sulphate, tricalcium aluminate and tantalum oxide. The latter serves as a radiopacifying agent, replacing bismuth oxide to prevent discoloration. Mixed with a water-based gel for a good handling, it releases a higher amount of calcium and presents a higher mineralization potential when compared to its predecessor NeoMTA Plus® (3).

In cases where endodontic treatment fails, a non-surgical retreatment can be performed with a success rate of about 80% (4).

Currently, nickel-titanium rotary instruments are used to remove gutta-percha and cement, reducing the time needed to reach working length and to remove most of the material. However, no technique can completely remove gutta-percha and sealant (5).

In both endodontic treatment and retreatment, achieving apical patency is crucial for infection control, reducing the number of microorganisms and preventing the accumulation of dentine debris in the final millimeters of the root canal.

During instrumentation and removal of root canal filling material, the extrusion of dentine debris, pulp tissue, microorganisms and/or irrigants into the periradicular tissues is inevitable (6).

Given the use of continuous and reciprocating nickel-titanium rotary instruments for endodontic retreatment and the application of calcium silicate hydraulic sealants, we formulated the following null hypothesis: there are differences in retreatment procedures depending on the rotation system used (continuous rotation (D-Race) or asymmetric reciprocation (Reciproc®)), and the type of sealer cement employed (epoxy resin (AHPlus™) or calcium silicate hydraulic cement (NeoMTA 2®)), considering debris extrusion, time to reach patency and retreatment time.

## Material and Methods

-Sample selection:

The protocol of this study was approved by the Bioethics Commission of the University of Barcelona.

A total of 60 mature monoradicular human teeth with a single straight canal, identified by digital radiographs, were selected for this study and kept in saline solution at 4ºC. Teeth with previous endodontic treatment, root caries, external and/or internal resorption, monoradicular teeth with curvatures and with more than one root canal and apical foramen were excluded.

-Root canal preparation and filling.

The entire procedure was conducted by a single operator using the Zeiss Opmi microscope (Carl Zeiss Meditec AG, Jena, Germany) at x5 magnification. To standardize the working lengths, the crowns were sectioned to obtain a root length of 19 mm. Nº 2 round diamond burs (Dentsply) were employed for the openings. Patency was confirmed by inserting a #10 K-file (Dentsply) through the apical foramen, and the working length (WL) was determined by subtracting 1 mm. The canals were instrumented with Race #35.06 (FKG), allowing a maximum of 5 canals per file.

A 5 ml of 2.5% NaOCl (Coltene, Alstätten, Switzerland) with a 30G needle (Hager & Werken, Duisburg, Germany) was used to irrigate. Canals were irrigated after each instrument and, after chemical-mechanical preparation, the intra-canal smear layer was removed with a final irrigation protocol: 2 ml of 17% EDTA solution for 2 min, 1 ml of 2.5% NaOCl for 30 s, and 1 ml of sterile saline for 30 s (11). Root canals were then dried with #35 sterile paper tips (Dentsply).

-Experimental design:

Before the application of the sealer cement, the teeth were randomly divided into two groups: 30 teeth were filled with AH Plus™, and the remaining 30 teeth were filled with Neo-MTA 2®. Both cements were prepared following the manufacturer’s instructions. All canals were filled following the single cone technique, with a #35 gutta-percha cone (Dentsply). The access cavities were then sealed with Cavit™ (3M ESPE, Neuss, Germany), and the quality of the intra-canal filling was verified through digital radiographs.

In cases where the filling was found to be deficient, these samples were replaced with new ones. The teeth retained for the remainder of the study were then stored in a humidifying chamber (maintaining 100% humidity at 37°C) for two weeks.

-Root canal retreatment:

The obturated teeth were divided into 4 experimental subgroups of 15 teeth each (n=15), based on the sealer cement and retreatment instrument. The retreatment process was carried out by the same operator.

The subgroups were as follows: group 1 = AH plus + Reciproc; group 2 = AHplus + D-Race; group 3 = NeoMTA2 + Reciproc and group 4 = Neo-MTA2 + D-Race.

Groups 1 and 3 were unfilled with a Reciproc R25 using the VDW Gold motor. Additional apical flaring was performed with an R50. For groups 2 and 4, the D-Race system was employed. A DR1 (#30; 10%) was initially used at to remove material from the coronal third, and a DR2 (#25; 4%) for the middle and apical third. Additional apical flaring was performed with a Race EVO 50, to standardize the apical diameter to 50 in both rotation systems. Each canal was irrigated with 2.5% NaOCl after each instrument. No solvent was used to soften the gutta-percha.

A total of 20 ml of 2.5% NaOCl was used during each retreatment, with 15 ml during the basic retreatment procedure and 5 ml during additional apical widening (7).

After completing retreatment, the final irrigation protocol described above was executed (7). Finally, the root canals were dried with paper points.

**Figure 1:**
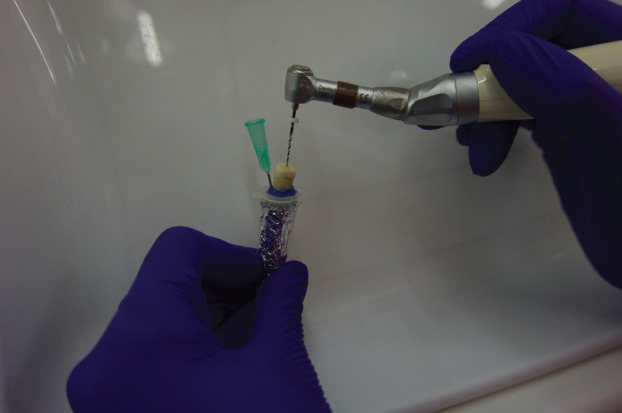
Non-surgical retreatment of monoradicular tooth *in vitro*.

-Retreatment time and collection of extruded debris:

The method employed to collect extruded debris followed the procedure proposed by Myers and Montgomery (8). Each tooth was inserted into an Eppendorf tube, with holes created in the rubber stoppers of the vials using a heated instrument. The tooth was pressed through the rubber stopper and fixed at the level of the cemento-enamel junction with LC Block-Out Resin® (Ultradent, Utah, USA). A 27-gauge needle (Hager & Werken, Duisburg, Germany) was positioned beside the plug to balance inner and outer pressure. The tubes were then placed in vials covered with aluminum foil to prevent the operator from seeing debris extrusion during the process (Fig. 1). The Eppendorf tubes were pre-weighed with an electronic balance (Analytical balance ABT KERN & SOHN GmbH, Germany) with an accuracy of 0.00005g. Three consecutive measurements were taken for each tube and the mean value was recorded.

The total retreatment time was measured and recorded in minutes and seconds using a digital stopwatch. The time measurement started when beginning the rotary instrumentation and ended when the last instrument reached WL, with no visible material on the file and the canal walls clear of microscopically visible debris. The time taken to achieve apical patency was also recorded.

Following retreatment, each tooth was removed from the Eppendorf tube and the root was washed with 1 ml of distilled water to collect any debris that may have adhered to the root surface. The tubes were subsequently stored in an incubator at 70°C for five days to evaporate distilled water and irrigant residues before weighing the dried debris. The same analytical balance was used to obtain the final weight of the tubes, including the extruded debris. Each tube was weighed three times and the mean value was calculated. The dry weight of the debris was determined by subtracting the weight of the empty tube from the weight of the tube with extruded debris.

-Statistical analysis

Descriptive analysis of the variables was conducted by calculating means and standard deviations for each study group. The normality of the variables was assessed using the Kolmogorov-Smirnov test. Population comparisons were executed using Student t-test for independent data. Mean differences were estimated with a 95% confidence interval. Hypothesis tests were performed with an alpha risk of 5%. Analyses were carried out with the JAMOVI 1.16.15 application, and the significance level was set at *P* = 0.05.

## Results

-Results according to cement type.

To evaluate potential differences in the variables “retreatment time”, “time to reach patency” and “debris weight” based on the type of cement, a comparison of means was conducted (Table 1), and a Student t-test was performed.

**Table 1:**
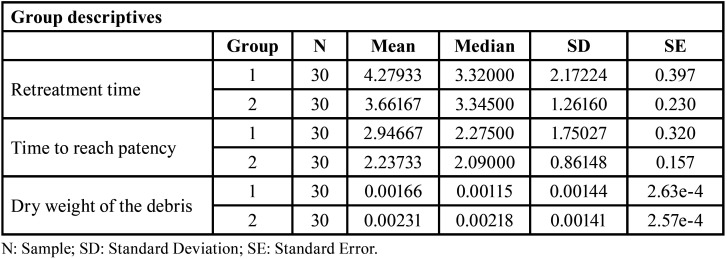
Descriptive variables according to cement type. Group 1: AHPlus™; Group 2: NeoMTA 2®.

No significant differences were observed for the 3 variables (*P* > 0.05) (Table 2), indicating that the hypothesis asserting equality between the 2 types of cement cannot be rejected.

**Table 2:**
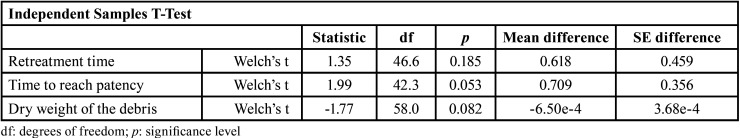
t-Student tests for independent data according to cement type.

-Results according to rotation system type used for retreatment.

To evaluate potential differences in the variables concerning the rotation system, the same analysis was conducted (Table 3).

**Table 3:**
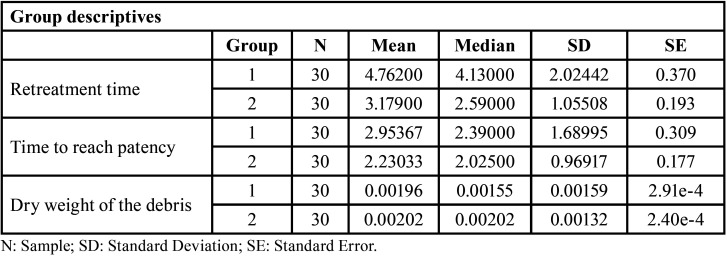
Descriptive of the variables according to the rotation system. Group 1: Reciproc, Group 2: D-Race.

**Figure 2:**
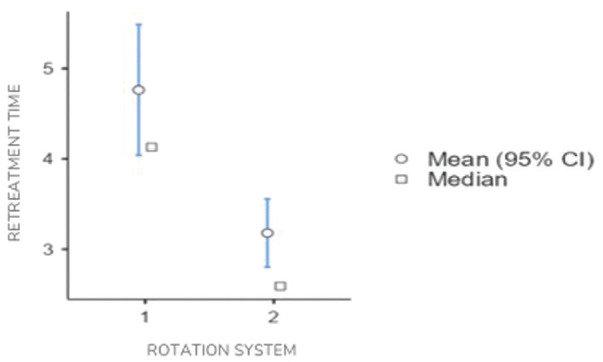
Mean plots with 95% confidence intervals for the variable “Retreatment time” for rotation systems 1 (Reciproc) and 2 (D-Race).

**Figure 3:**
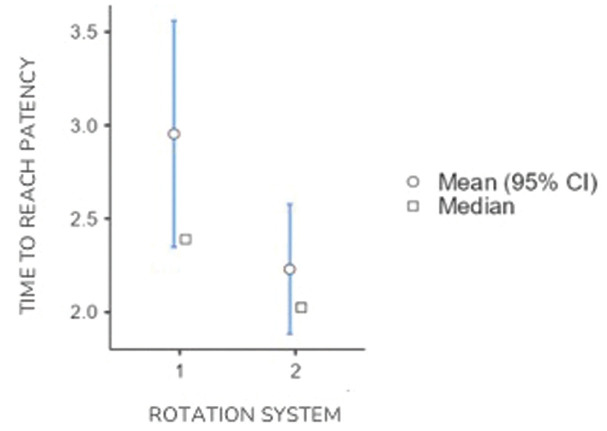
Mean plots with 95% confidence intervals for the variable “Time to reach patency” for rotation systems 1 (Reciproc) and 2 (D-Race).

In this case, “retreatment time” and “time to reach patency” exhibited statistically significant differences (*P* < 0.05), while “debris weight” did not demonstrate statistically significant differences (*P* > 0.05) (Table 4, Figs. 2,3).

**Table 4:**
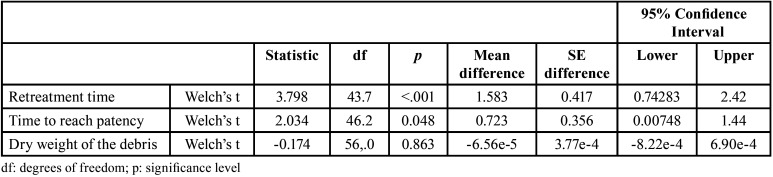
Student t-tests for independent data depending on rotation system.

The present study revealed that, on average, more time was required to remove the filling material in group 1 (Reciproc) compared to group 2 (D-Race), with a statistically significant mean difference of 1.59 minutes.

## Discussion

Extruded debris can lead to postoperative complications such as pain and swelling. It is important to use techniques that minimize apical extrusion (9,10).

Huang *et al*. (11) reported significantly less apical extrusion with ProTaper Universal (Dentsply) compared to hand instrumentation.

In contrast, Somma *et al*. (12) found that Hedström files produced less debris extrusion than Mtwo R retreatment files (VDW) and ProTaper.

Mollo *et al*. (13) evaluated extruded material during retreatment with two rotary systems, Mtwo R and R-Endo (Micro-Mega, Besanςon, France), and hand files. They observed that the R-Endo system caused less apical extrusion than hand files.

Topçouglu *et al*. (14) observed less extrusion with ProTaper, D-RaCe and R-Endo rotary retreatment instruments compared to hand files. Similarly to our study, there was no significant difference between the rotary systems.

In retreatments, comparing rotary and hand files consistently shows that hand files tend to result in a higher level of debris extrusion (11,13,14).

In a recent systematic review, Caviedes *et al*. (15) concluded that the alternative systems (WaveOne (Dentsply) and Reciproc (VDW, Munich, Germany)) tend to extrude more material in the periapical tissues than continuous rotation systems such as Mtwo retreatment system (VDW) and Protaper Universal retratment (Dentsply). While our results differ from this conclusion, it’s important to note the absence of a direct comparison between Reciproc and D-Race in this study.

As a bioceramic cement we used NeoMTA 2®, which has good sealing ability (3) but could make its removal difficult during retreatment.

The achievement of working length (WL) and patency in retreatment cases holds great importance to improve periapical healing rates (16). In the present study, WL was achieved in 100 % of the samples. In contrast, Baranwal *et al*. (17), which employed the ProTaper Universal retreatment system and the BioRootTM RCS sealer, reported achieving apical patency in 80% of the specimens.

Eymirli *et al*. (18) claimed to be able to reach WL if gutta-percha was used together with EndoSequence BC Sealer®, while they were unable to achieve it in samples filled exclusively with sealer.

Athkuri S. *et al*. (19) reported that retreatment time was not significantly affected by the type of sealer (AHPlusTM and BioRootTM RCS) but by the filling technique; they observed that retreatment time was shorter with lateral compaction technique compared to vertical compaction and thermoplasticized injectable techniques.

Further studies are needed to evaluate the re-treatability of calcium silicate sealers using different endodontic instruments and different filling techniques.

## Conclusions

The results of this *in vitro* study indicated that all systems used in retreatment cause apical extrusion of debris regardless of the file system and sealer cement used, with no statistically significant differences (*P* > 0.05). In terms of time to reach patency and retreatment time, the time with the Reciproc file system was longer compared to the D-Race file system, with statistically significant differences (*P* < 0.05). Apical patency was achieved in all cases.
